# Factors Influencing Adherence to Phototherapy in Patients With Psoriasis: A Cross‐Sectional Study

**DOI:** 10.1111/jan.16472

**Published:** 2024-09-24

**Authors:** Elena Violeta Iborra‐Palau, Elena Garcia‐Redondo, Raquel Alabau‐Dasi

**Affiliations:** ^1^ Department of Nursing, School of Nursing and Podiatry University of Valencia Valencia Spain; ^2^ General University Hospital of Valencia Valencia Spain; ^3^ Malvarrosa Hospital Valencia Spain; ^4^ Department of Nursing and Podiatry, School of Health Sciences University of Malaga Malaga Spain

**Keywords:** adherence, phototherapy, psoriasis, quality of life and nursing

## Abstract

**Aims:**

Assess the level of adherence to phototherapy and determine what factors influence it.

**Design:**

Cross‐sectional.

**Methods:**

This study included a convenience sampling of 72 patients with psoriasis undergoing phototherapy. Data were collected through a self‐reported questionnaire with sociodemographic variables, the Goldberg Anxiety and Depression Scale, the Short Form Health Survey and the Dermatology Life Quality Index. Adherence to the treatment and its ending was measured through a session record.

**Results:**

A small percentage of the participants demonstrated adequate adherence, and nearly half of them had low adherence. The factors statistically significant and with a negative impact on adherence were as follows: having a partner, experiencing anxiety or depression or using public transportation to get to the hospital. The probability of not adhering to the treatment increased when: patients found it difficult to attend therapy; perceiving their mental and physical health as being worse; experiencing anxiety or depression; having a diagnosed mental illness; being a man; or having had the sickness for an extended period of time.

**Conclusion:**

This study determined the level of adherence to phototherapy and advanced our understanding of this variable. Women exhibited higher levels of adherence compared to men, although they reported worse perceived mental and physical health, and the disease had a higher impact on their life.

**Implications for the Profession and/or Patient Care:**

Informing phototherapy nurses on the factors that impact treatment adherence may help to increase the treatment compliance, which may improve psoriasis patients' clinical symptoms.

**Impact:**

Increase the body of knowledge about the treatment that phototherapy nurses administer.

**Reporting Method:**

STORBE guidelines.

**Patient or Public Contribution:**

No patient or public contribution.

**Summary**

**What Does This Paper Contribute to the Wider Global Clinical Community?**

- Identifying key factors influencing phototherapy adherence in psoriasis patients.
- Improving treatment adherence in patients with skin conditions.
- Increasing nursing knowledge regarding phototherapy.

## 1 Introduction

Psoriasis has been considered as a dermatological disease characterised by inflammation, chronicity and being immune‐mediated (Mrowietz, Steinz, and Gerdes 2014). This disease's aetiology is complex, involving both genetic predisposition factors and environmental triggers (Harden, Krueger, and Bowcock 2015). The pathophysiology of this dermatosis has been described as an immune imbalance with a significant inflammatory component mediated by T cells (Langrish et al. 2005). Furthermore, as a result, people with psoriasis are more likely to develop a number of concomitant diseases and comorbidities (Pupo et al. 2019; Mrowietz, Steinz, and Gerdes 2014). In fact, cutaneous symptoms can be caused by a variety of factors, including trauma (Sagi and Trau 2011), medications (García and Baca‐García 2002), toxic habits like tobacco and alcohol (Gerdes et al. 2010), stress and anxiety (Rousset and Halioua 2018).

Also, this disease is widely extended worldwide among the general population (Michalek, Loring, and John 2017). Although the prevalence of this disorder might range, from 0.51% (Takeshita et al. 2015) to 11.43% (Danielsen et al. 2013) in specific areas and for different reasons. Michalek, Loring, and John (2017), in their systematic review, identified that the overall prevalence of psoriasis in adults is nearby 3%. For example, the average prevalence in Denmark is 3.73%, 3.10% in Italy and 3.9% in Sweden. Moreover, the severity of the outbreaks of psoriasis affects the quality of life for those who suffer from this condition, leaving them with a heavy emotional burden as a result (Gupta and Gupta 2003). Furthermore, the psychological comorbidity persists even after the skin symptoms disappear (Madrid Álvarez 2018). For example, Laguna, Payero, and Márquez (2006), who measured the anxiety levels of 132 individuals with different dermatological diseases, found that patients with psoriasis had higher levels of anxiety than those with other dermatoses, with the exception of atopic dermatitis. On the other hand, it is known that this disease can be caused by prolonged anxiety and its effect on the immune system (Daschuk, Dobrzhanskaya, and Pustovaya 2018). This creates a vicious circle from which it is difficult to break free without professional help.

Although there are no definitive treatments for this disease, there are treatments that can alleviate symptoms. One of these treatments is phototherapy, which involves projecting ultraviolet radiation onto the patient's body surface, and its benefits have been extensively studied (Hönigsmann 2016). When a person's health does not allow for the prescription of systemic or biological treatments, this treatment has been shown to be extremely helpful (Calzavara‐Pinton et al. 2018). Despite its benefits, it requires a strong commitment and consistent attendance from the patient. They must visit the hospital 2–3 times per week, and the positive effects on the patient's skin take several weeks to show (Carrascosa et al. 2005). This last fact could have a significant impact on their adherence to the treatment.

## 2 Background

The academic literature emphasises the importance of maintaining elevated levels of adherence for the treatment to be effective (Brownell, Wang, and Tsoukas 2016). However, people with chronic dermatoses are frequently disengaged from their treatments (Maibach 2006). For example, in a retrospective study, Weng et al. (2016) discovered that adherence was < 70%, whereas Leal Canosa (2013) discovered that non‐adherence rates for phototherapy reached 88%. Furthermore, in a study of 851 psoriasis patients receiving phototherapy in a hospital, 53% of the sample did not adhere effectively, and one third attended fewer than 10 treatment sessions (Kalia et al. 2014). Even phototherapy treatments at home suffer from low adherence rates (Cline et al. 2019). Nonetheless, none of these studies provide a clear answer to the question of what level of phototherapy adherence is considered appropriate. This is due to the significant variation in how adherence was measured across all of the studies reviewed (Cline et al. 2019; Evers et al. 2010; Kalia et al. 2014; Weng et al. 2016). Furthermore, there is a lack of evidence identifying the factors that influence patient non‐adherence (Maibach 2006; Torrelo et al. 2013). However, the few studies that exist yield few interesting conclusions. For example, the lack of public transportation and the distance that patients must travel to the hospital to receive treatment have a significant impact on adherence to therapy (Brownell, Wang, and Tsoukas 2016; Kalia et al. 2014; Weng et al. 2016). In addition, Evers et al. (2010) discovered that the severity of the disease correlates with lower adherence to phototherapy. Leal Canosa (2013) also noticed that patients who saw their dermatologist more frequently had better adherence rates.

In contrast, despite the fact that the nurse supervises, administers the phototherapy service and interacts with the patient several times per week, the adherence‐related literature reviewed in this study is focused on investigations conducted by doctors and dermatologist. This aspect ignores the nursing perspective on the problem, preventing it from being improved holistically. The aim of this investigation was to provide other nurses with a starting point from which to intervene and improve phototherapy adherence rates.

## 3 The Study

### 3.1 Aims

To determine the factors that influence adherence to phototherapy in patients with psoriasis.

## 4 Methods

### 4.1 Study Setting and Sampling

This study was conducted between 2019 and 2022 at the University General Hospital of Valencia's phototherapy service in Spain. The sample was obtained using non‐probabilistic convenience sampling. Finally, 72 participants who met the inclusion criteria were included and analysed. These criteria were as follows: (a) having psoriasis; (b) being over the age of 18 years; and (c) receiving phototherapy with a wavelength of ultraviolet type UVB narrow‐band in this service. To participate in this study, the participants were required to sign an informed consent form. On the other hand, this study excluded people with other dermatoses, as well as those who lacked cognitive capacity or did not understand the survey's language.

### 4.2 Ethical Considerations

This study's design and procedure followed the Declaration of Helsinki. Ethical approval was obtained from the Ethics Committee of the University General Hospital of Valencia, in Valencia, Spain. With the approval number 07/2019, on 7 November 2019. Written informed consent was obtained from each participant who agreed to participate in this study. The participants' right to withdraw from the study at any time had no detrimental effects on their routine treatment, care or other services.

### 4.3 Data Collection

A survey was used to gather information on sociodemographic characteristics, means of transportation and ease of attendance at therapy. Additionally, each patient's adherence level and outcome at the end of treatment were evaluated. Validated scales were used to collect information about people's health status. The instruments used to measure the variables are then described. There were no missing data points, and every participant answered every survey question.

#### 4.3.1 Goldberg Anxiety and Depression Scale (GADS)

The Goldberg Anxiety and Depression Scale (GADS) was used to assess patients' perceived mental health. This scale was used to determine whether anxiety, depression or both existed. The questionnaire is divided into two subscales: one for anxiety and one for depression, each consisting of nine questions with dichotomous answers. Each time a participant answers ‘yes’ to a question, he or she receives one point. When the patient answers four or more questions affirmatively on the corresponding subscale, he or she exhibits anxiety symptoms. Compatible symptoms of depression are defined when answers affirmatively to two or more questions on the corresponding subscale (Monton et al. 1993).

#### 4.3.2 Short Form Health Survey

The Short Form Health Survey SF‐12 (SF‐12) was used to determine the patients' perceived health status. This instrument, derived from the SF‐36 Health Survey, was designed to reduce administration time. It is made up of 12 items, divided into physical and mental categories (Ware Jr., Kosinski, and Keller 1996). The average score on this questionnaire for the general population is 50 points. When the obtained score is less than this threshold, it indicates that the individual perceives being less healthy than the general population (Vilagut et al. 2008).

#### 4.3.3 Dermatology Life Quality Index (DLQI)

This instrument, designed specifically for people suffering from dermatological diseases (Finlay and Khan 1994), was used to assess how the disease affected the participants' quality of life. It consists of 10 questions that are answered using a Likert‐type scale with scores ranging from 0 to 3. The final score can range from 0 (no impact on quality of life) to 30 points (extremely large effect on patient's life). Scores are categorised as follows: no effect at all of patient's life (0–1), small effect on patient's life (2–5), moderate effect on patient's life (6–10), very large effect on patient's life (11–20) and extremely large effect on patient's life (21–30) (Hongbo et al. 2005).

#### 4.3.4 Level of Adherence to Phototherapy

The adherence level was calculated by comparing the sessions attended by the individual with those, considering the temporal context in which they occurred. This way, we obtained a percentage of completed sessions. Once the percentages were determined, and considering the high rates required for the therapy to be effective, the variables were categorised into three levels of adherence: (1) adequate, with more than 90% of sessions completed; (2) moderate, with 85%–89% of sessions completed; and (3) low, with < 84% of sessions completed. Additionally, the treatment's outcome for each patient was recorded. This variable considered whether the person abandoned the treatment, was medically discharged with resolution of the outbreak or ended the treatment due to changing therapies. These associations allowed us to establish relationships between adherence levels and individual outcomes to determine the effectiveness of the treatment.

#### 4.3.5 Sociodemographic Variables and Other Variables of Interest

These variables included: age, gender (male and female), marital status (single, married, divorced, widowed and stable partner), employment (employed, unemployed, retired and student), dependents (children, grandchildren, mother/father or someone older, several and none), comorbidities (diabetes, cardiovascular disease, hepatic disease, oncologic disease, several and none), mental illness diagnosed by a psychiatrist (yes and no), ease of attending to therapy (yes and no), transportation to attend to therapy (walking, bus or train same city, bus or train different city, car and has to be brought by someone), financial effort to attend to therapy (Likert scale of 1–5 points, from low effort until maximum effort), diagnosed pathology (psoriasis all over the body and palmar‐plantar psoriasis) and duration of disease (more than 5 years and < 5 years).

### 4.4 Data Analysis

First, a descriptive analysis was performed on all the variables of the study, disaggregating them by gender. To evaluate whether the differences discovered between the ‘gender’ variables were statistically significant, different statistical analysis techniques were used for each type of variables. When gender was crossed with categorical variables, the Chi‐square test was used, and when frequency of the categories was < 5, the Fisher test was used. When gender was crossed with numerical variables, the Student's *t*‐test for independent samples was used. The choice of parametric test was based on earlier verification of the sample's normal distribution using Kolmogorov–Smirnov normality test in each case. Subsequently, the odds ratio (OR) was performed to observe the probability of not being adherent versus being adherent for each group of dichotomic variables. Finally, a simple linear regression was implemented for all predicting variables to explain the response to variable ‘adherence’. For all this statistical data analysis, the software used was ‘R’ (R Core Team 2018), and *p* < 0.05 were considered statistically significant.

## 5 Results

### 5.1 Characteristics of Participants

Primarily, all participants met the criteria for the data analysis stage. Of the 72 subjects, 57% were women and 43% were men, with an average age of 46 years. Palmar‐plantar psoriasis affected 22.2% of the participants, while the remaining 77.8% had psoriasis elsewhere on their bodies. Regarding pathology's chronicity, 72% of the sample had the disease for more than 5 years. Women were diagnosed with mental illness at a rate of 15.3%, compared to 8.3% for men. Furthermore, 20% of the patients reported difficulty attending treatment, with only female patients stating that they had to make significant or extreme financial efforts to attend treatment. In the case of women, 2.8% needed to be transported to the hospital, whereas men did not need anyone to take them there. Also, 29.2% of participants did not have a partner because they were divorced, widowed or single, while 70.8% were married or had a stable relationship. Regarding employment status, 58.3% of the sample was active, 25% were retired and the remainder were unemployed (12.5%) or students (4.2%). Women had the most family responsibilities (57% vs. 43% for men).

### 5.2 Quality of Life (DLQI), Health Status (SF‐12), Anxiety and Depression (GADS) Before and After the Treatment

When asked about the disease's impact on quality of life, measured with the DLQI, only 6.9% said it had no effect at all on them, while 26.3% said it had a small effect on their life. On the other hand, 29.1% reported a moderate effect, 33.3% very large effect and 4.1% that it had an extremely large effect. The perceived health status, measured by the SF‐12, including both physical and mental components, was poorer than the overall population's perceived health. In terms of the physical component of perceived health, men scored 48.6 points while women scored 45.7. The mental component of perceived health weakened for both genders. Men scored an average of 45.8, while women scored 41.2. The GADS identified an elevated incidence of anxiety‐depressive symptoms. Seventy‐five per cent of the participants had anxiety, while 48.6% of them had depression. When broken down by gender, women had higher levels of anxiety and depression than men (see Table 1). Overall, women had worse health than men, but only the DLQI showed a statistically significant gender difference in scores (*p* = 0.012).

**TABLE 1:** Percentage values of the presence of anxiety and depression calculated over the total number of people of each gender.

Gender	Anxiety (%)	Depression (%)
Male	70.9	38.7
Female	78	56

### 5.3 Therapeutic Adherence Phototherapy

The overall average adherence rate was below 83.2% of the sessions conducted, where women had an average of 83.9% of sessions and men had 82.2%. However, to consider moderate or adequate adherence, the values of the sessions conducted had to be between 85% and 89% or above 90%, respectively. It was found that 54.7% of the patients had low adherence, 21.9% had moderate adherence and 23.4% had adequate adherence. Table 2 presents the adherence data disaggregated by gender, revealing that women had greater levels of adherence. In terms of therapeutic outcomes, 52.8% of the sample was discharged with the outbreak completely resolved, 38.9% required a change in treatment and 8.3% dropped off. Also, in this case, women had a greater rate of medical discharge (53.4%) than men (48.3%). When the degree of adherence was analysed alongside the outcome of treatment, it became clear that people who changed treatment or dropped it had lower average adherence (77.5% and 78%, respectively) than people who successfully resolved the outbreak and were discharged from the hospital (88.2% adherence).

**TABLE 2:** Percentage values of the categorised adherence variable calculated over the total number of people of each gender.

Gender	Low (%)	Moderate (%)	Adequate (%)
Male	58.1	12.9	29
Female	43.9	22	34.1

#### 5.3.1 Odds Ratio of Non‐Adherent Versus Adherent

To determine the odds ratio, the adherence variable was transformed to a dichotomous one. A group was created for people considered non‐adherent, with those who had a low degree of adherence. Another group of patients considered adherents were those who had a moderate or adequate degree of adherence. The categories where there was a greater probability of not being adherent were as follows: being a man (1.38 (0.80–2.38)), having a diagnosed mental illness (1.13 (0.49–2.59)), not being able to go to therapy easily (1.14 (0.46–2.82)), suffering from anxiety (1.35 (1.02–1.78)), suffering from depression (1.69 (1.02–2.81)), suffering from psoriasis vulgaris (1.06 (0.84–1.38)), suffering from the disease for more years (1.08 (0.81–1.44)) or having sick leave. perceived health, both physical (1.11 (0.74–1.66)) and mental (1.38 (1–1.90)).

#### 5.3.2 Relationship of Predictor Variables and Response Variables ‘Adherence’

Using simple linear regression, it was possible to verify that the study's independent variables influenced the dependent variable ‘adherence’. When conducting this analysis, it was discovered that the following variables had a negative impact on adherence: the presence of anxiety (*p* = 0.025), the presence of depression (*p* = 0.014), taking public transport, whether in the same city (*p* = 0.03) or from another town (*p* = 0.021) and having a stable partner (*p* = 0.001). On the other hand, it was found that having an oncological disease could improve adherence (*p* = 0.007).

## 6 Discussion

The findings identified in this study are consistent with other scientific papers in that adherence is a crucial aspect of the success of a treatment. According to the findings of Barros et al. (2021) and Brownell, Wang, and Tsoukas (2016), phototherapy needs an elevated level of adherence for it to be effective. In this study, the effectiveness of phototherapy was collected by annotating the patient's outcome when finishing the treatment. It showed that slightly more than half of the participants were discharged after the outbreak was under control, while the remaining participants (38.9%) had to switch treatments or stop it altogether (8.3%) due to a lack on their improvement. By contrasting the results, it was concluded that non‐adherence may be one of the reasons for not seeing clinical progress. The group of patients who abandoned or changed treatment had a lower percentage of sessions completed than the sample who were discharged. It would be reasonable to think that there were enough sessions completed in the group of patients who changed treatments (77.5% of completed sessions) or dropped out (78% of completed sessions). Despite the fact that these patients attended over 77% of the sessions, the medical professional failed to discharge them.

A review of the literature was conducted to investigate the criteria proposed by other authors for measuring phototherapy adherence in a standardised manner. However, there was no consensus on how to measure this concept. For example, Kalia et al. (2014) and Weng et al. (2016) established different cut‐off points for defining if someone was ‘adherent’ or ‘non‐adherent’. The first authors used 20 sessions, while the later used six sessions. Moreover, Cline et al. (2019) compared the actual number of sessions each patient received with the number of sessions they had the opportunity to receive during the therapy period. On the other hand, Evers et al. (2010) did not measure adherence based on the number of sessions completed. Instead, patients were asked about their perceived adherence to the treatment at distinct levels using a Likert‐type scale. This is why, it has been challenging to reliably contrast the results obtained with those of other studies due to the lack of homogeneity in the way adherence was measured. Nonetheless, it is worth reflecting on the results obtained by other authors and contrasting them as much as possible with those of this research. For example, Cline et al. (2019) observed that the average adherence in their sample of psoriasis patients undergoing phototherapy was 80%. When disaggregating the data by gender, it obtained lower figures for men (79.3%) than for women (82%). These results are remarkably similar to those from this study, where the average was slightly larger (83.2%), and men (82.2%) were less adherent than women (83.9%). Kalia et al. (2014) and Weng et al. (2016) used a dichotomous approach to quantify adherence (adherent vs. non‐adherent). The former had a 68.1% adherence rate, while the latter had 47%. It should be noted that, in this study, adherence was also recoded as a dichotomous variable to determine the odds ratio. This allowed us to compare our findings to those of prior authors. In this situation, adherence was 45.3%, which is more similar to the findings of Kalia et al. (2014). Disaggregating the data by gender, this study found that women were still more adherent (56.1%) than men (41.9%). Moreover, Weng et al. (2016) disaggregated data by gender (59.2% for women and 40.8% for men) and obtained similar data to the one in this study.

Other examples of the lack of consensus, was the minimum number of phototherapy sessions that should be performed for a participant to be considered adherent. For instance, in the case of Kalia et al. (2014), less than six sessions were deemed to be ‘non‐adherence’, whereas in the case of Kalia et al. (2014) and Weng et al. (2016), the cut‐off was less than 20 sessions. Nevertheless, Cline et al. (2019) were the only authors to develop a method of measuring adherence similar to the one used in this investigation. However, 15 participants were excluded from their investigation for “not meeting the minimum adherence criteria.” Therefore, the significance of the study's findings might not be as transferable to other contexts if patients with insufficient compliance were dismissed. In spite of these differences, the degree of participant adherence to this investigation is consistent across all studies when comparing the variable in a longitudinal manner. Additionally, adherence was higher in women in the publications where data were separated by gender, as it is in this study. Therefore, if any progress is to be made in this field, it is imperative to obtain consensus on the way that adherence should be measured, as well as be conscious of the factors affecting it.

Despite the diversity of ways for measuring adherence, in all the contrasted studies, as in this study, the rates of adherence to phototherapy were insufficient. When examining the elements in the current investigation that affect the patient's adherence to phototherapy, one of the factors could be the location where the study was conducted. Valencia is a Spanish city near the Mediterranean, with a warm climate all year where it is possible to enjoy the beach and the sun (Gil‐Alana, Gil‐Lopez, and Román 2021). These conditions are extremely beneficial for the whitening of psoriasis disease and the reduction of its symptoms (López 2007). Given these conditions, it could be assumed that the participants of this study did not attend therapy because it was not needed. However, it has been shown that the non‐adherent patients were those who did not obtain medical discharge or did not have enough improvement so finally changed treatment or abandoned it. Therefore, further investigation should be done to analyse why the participants of this study could not enjoy the benefits of spending time outdoors. Moreover, regardless of the climate of the area, a multitude of factors have been found that could be negatively affecting adherence.

The current research differs from the few conclusions drawn from other studies regarding the factors influencing adherence to phototherapy. Although adherence was measured by Cline et al. (2019) and Yentzer et al. (2008), no variables that might have an impact on adherence were collected in any of these studies. According to Kalia et al. (2014), age and distance from the therapy location were the two factors strongly linked to non‐adherence. It was more challenging for their participants to adhere to the treatment when they were younger and having to commute longer distance to go to treatment. Weng et al. (2016) also found that adherence decreased with increasing distance, highlighting the need to investigate this variable as a factor influencing these patients' financial circumstances. In the case of this study, distance was not measured; however, patients' ways of transportation to and from treatment was recorded. The results indicated that using public transportation (i.e., bus or train) decreased adherence when compared to using one's own vehicle, walking or other independent means. These results imply that a patient's likelihood of adhering to a treatment plan is significantly influenced by socio‐economic factors, and more research is required to confirm this.

Other factors, which are frequently diagnosed in patients with dermatological disorders, are psychiatric disorders. For example, two studies examined this aspect in psoriasis patients (Tribo et al. 2019) and (Laguna, Payero, and Márquez 2006), and the prevalence of a psychiatric pathology in both studies was similar to the current investigation with 23.6% of participants presenting it. In one of them, the prevalence reported was 25% of the sample (Tribo et al. 2019), while in the second paper, it was 21% (Laguna, Payero, and Márquez 2006). Moreover, Tribo and her colleagues agreed with the current research in that women had greater prevalence of psychological comorbidity than men, both in the presence of anxious and depressive symptoms, as well as when suffering from mental illness. However, in terms of the impact on quality of life, men perceived a greater negative impact (Tribo et al. 2019), which differed from the findings of this study. On the other hand, Leal Canosa (2013) found no relationship between being less adherent and factors such as a psychological condition or quality of life in terms of health status. However, this paper has shown that the likelihood of not adhering to phototherapy could be influenced by depression, anxiety or having a low perceived physical health.

### 6.1 Strengths and Limitations

The fact that the study began shortly before the COVID‐19 pandemic limited the collection of data and the study's progress. The participants' therapy was suspended during lockdown, and the nurse in charge of the research was called in to assist in other services due to the exceptional situation. To avoid the bias that could arise from this fact, since the patients were locked down by government order and did not attend phototherapy, the sessions that were suppressed for this reason were not considered in the data analysis. Even so, this significant limitation may limit the extrapolation of this study, even after correcting the sessions calculation to determine adherence.

Another limitation could be the non‐existent information about the disease's chronicity and level of exacerbation on the patients. This is because all of the participants were experiencing a psoriasis outbreak at the time of the study and required phototherapy treatment. However, these variables could have been documented to better understand the patients' medical history and if past outbreaks had an impact on the current adherence to phototherapy.

### 6.2 Recommendations for Future Research

Phototherapy remains an underexplored therapy in terms of the factors that can influence it and the consensus on how to quantify it. To present, there are no universally accepted criteria for determining what constitutes appropriate adherence to achieve the success of this treatment. This point is critical for the ability to implement healthcare procedures that allow nurses to address the reasons that prevent patients from attending therapy. The recommendation is to conduct a multicentred study with the same methodology, and outside the context of a pandemic to reinforce and expand the findings of this paper.

## 7 Conclusion

Based on the results obtained, it can be concluded that most of the sample did not follow the recommended guidelines to the fullest extent possible in order to maximise treatment effectiveness. After searching for potential influences on this factor, we have concluded that further research into psychological, social or economic factors is required. Moreover, when dividing the participants by gender, they had different results regarding adherence, impact on quality of life, mental health status and perceived health status. Despite the fact that women presented a worse situation with respect to the aforementioned variables, they were more adherent to treatment than men. Therefore, it is determined that distinct strategies to improve adherence to phototherapy ought to be contemplated for men and women.

## Author Contributions

Made substantial contributions to conception and design, acquisition of data or analysis and interpretation of data: E.V.I.‐P. Involved in drafting the manuscript or revising it critically for important intellectual content: E.V.I.‐P., E.G.‐R., R.A.‐D. Given final approval of the version to be published. Each author should have participated sufficiently in the work to take public responsibility for appropriate portions of the content: E.V.I.‐P., E.G.‐R., R.A.‐D. Agreed to be accountable for all aspects of the work in ensuring that questions related to the accuracy or integrity of any part of the work are appropriately investigated and resolved: E.V.I.‐P., R.A.‐D.

## Ethics Statement

Study obtained ethical approval (no. 7/2019) from the Research Ethics Committee of the University General Hospital of Valencia at 7 November 2019.

## Conflicts of Interest

The authors declare no conflicts of interest.

### Peer Review

The peer review history for this article is available at https://www.webofscience.com/api/gateway/wos/peer‐review/10.1111/jan.16472.

## Supporting information


Data S1.

## Data Availability

Any data utilised in the submitted manuscript have been lawfully acquired in accordance with The Nagoya Protocol on Access to Genetic Resources and the Fair and Equitable Sharing of Benefits Arising from Their Utilization to the Convention on Biological Diversity. The statistics were checked prior to submission by an expert statistician, Lorena González‐Pozo (gonzalezpozolorena@gmail.com).
