# Supplementary material for: Factors Influencing Adherence to Phototherapy in Patients With Psoriasis: A Cross‐Sectional Study
Source: J Adv Nurs. 2024 Sep 24;81(6):3110–7. doi: 10.1111/jan.16472 (PMC12080098; doi:10.1111/jan.16472)
Supplement: Supplementary file 1 — Data S1. [file JAN-81-3110-s001.pdf]

STROBE Statement—checklist of items that should be included in reports of observational studies

	Item No.	Recommendation	Page No.	Relevant text from manuscript
**Title and abstract**	1	(*a*) Indicate the study's design with a commonly used term in the title or the abstract	1	Factors influencing adherence to phototherapy in patients with psoriasis and atopic dermatitis: a cross-sectional study.
		(*b*) Provide in the abstract an informative and balanced summary of what was done and what was found	1	
**Introduction**				
Background/rationale	2	Explain the scientific background and rationale for the investigation being reported	2 and 3	
Objectives	3	State specific objectives, including any prespecified hypotheses	2 and 3	It aims to clarify a valid and reliable method for measuring treatment adherence while also identifying the factors that may influence this variable. This is done to give other nurses a starting point from which to intervene and try to improve phototherapy adherence levels
**Methods**				
Study design	4	Present key elements of study design early in the paper	4	
Setting	5	Describe the setting, locations, and relevant dates, including periods of recruitment, exposure, follow-up, and data collection	4	The study was conducted between 2019 and 2022 at the phototherapy service of the University General Hospital of Valencia, Spain
Participants	6	(*a*) *Cohort study*—Give the eligibility criteria, and the sources and methods of selection of participants. Describe methods of follow-up<br>*Case-control study*—Give the eligibility criteria, and the sources and methods of case	4	Finally, a total of eighty-four people who met the inclusion criteria were included in the

		ascertainment and control selection. Give the rationale for the choice of cases and controls<br>***Cross-sectional study*—Give the eligibility criteria, and the sources and methods of selection of participants**		sample. These criteria were: (a) suffering from psoriasis or atopic dermatitis; (b) being of legal age; and (c) receiving phototherapy in this service. These people had to sign the informed consent form to participate.
		(*b*) *Cohort study*—For matched studies, give matching criteria and number of exposed and unexposed<br>*Case-control study*—For matched studies, give matching criteria and the number of controls per case		
Variables	7	Clearly define all outcomes, exposures, predictors, potential confounders, and effect modifiers. Give diagnostic criteria, if applicable	4	Those people with dermatoses other than those mentioned and who did not have the cognitive capacity or who did not understand the language of the surveys were excluded from the study.
Data sources/ measurement	8*	For each variable of interest, give sources of data and details of methods of assessment (measurement). Describe comparability of assessment methods if there is more than one group	5 to 7	
Bias	9	Describe any efforts to address potential sources of bias	-	
Study size	10	Explain how the study size was arrived at	4	The duration of the contract of the first author with the hospital determined the sample size, more participants could not be added due to its expiration.
Quantitative variables	11	Explain how quantitative variables were handled in the analyses. If applicable, describe which groupings were chosen and why	5 to 7	
Statistical methods	12	(*a*) Describe all statistical methods, including those used to control for confounding	7	
		(*b*) Describe any methods used to examine subgroups and interactions	7	

		(*c*) Explain how missing data were addressed	5	No missing data was observed, all participants answered every question of the surveys
		(*d*) *Cohort study*—If applicable, explain how loss to follow-up was addressed<br>*Case-control study*—If applicable, explain how matching of cases and controls was addressed.<br>***Cross-sectional study*—If applicable, describe analytical methods taking account of sampling strategy**	7	Finally, a statistical model of multiple regression was implemented for all predicting variables to explain the response variable "adherence." For all this statistical data analysis, the software used was "R" (R Core Team, 2018), and p values <.05 were considered statistically significant.
		(*e*) Describe any sensitivity analyses	-	
**Results**				
Participants	13*	(a) Report numbers of individuals at each stage of study—eg numbers potentially eligible, examined for eligibility, confirmed eligible, included in the study, completing follow-up, and analysed	8	Of the total sample, all of them qualified for the data analysis stage. 57% were women and 43% were men.
		(b) Give reasons for non-participation at each stage	-	
		(c) Consider use of a flow diagram	-	
Descriptive data	14*	(a) Give characteristics of study participants (eg demographic, clinical, social) and information on exposures and potential confounders	8	
		(b) Indicate number of participants with missing data for each variable of interest	-	
		(c) *Cohort study*—Summarise follow-up time (eg, average and total amount)	-	
Outcome data	15*	*Cohort study*—Report numbers of outcome events or summary measures over time		
		Case-control study—Report numbers in each exposure category, or summary measures of exposure		
		***Cross-sectional study*—Report numbers of outcome events or summary measures**	Table 1 to 5	
Main results	16	(*a*) Give unadjusted estimates and, if applicable, confounder-adjusted estimates and their precision (eg, 95% confidence interval). Make clear which confounders were adjusted for	Table 6	

		and why they were included		
		(*b*) Report category boundaries when continuous variables were categorized	-	
		(*c*) If relevant, consider translating estimates of relative risk into absolute risk for a meaningful time period	-	
Other analyses	17	Report other analyses done—eg analyses of subgroups and interactions, and sensitivity analyses	-	
**Discussion**				
Key results	18	Summarise key results with reference to study objectives	10 to 12	
Limitations	19	Discuss limitations of the study, taking into account sources of potential bias or imprecision. Discuss both direction and magnitude of any potential bias	13	The fact that the study began shortly before the COVID-19 pandemic sometimes limited the collection of data and the study's progress. The participants' therapy was suspended during lockdown, and the nurse in charge of the research was called in to assist in other services due to the exceptional situation.
Interpretation	20	Give a cautious overall interpretation of results considering objectives, limitations, multiplicity of analyses, results from similar studies, and other relevant evidence	13 and 14	
Generalisability	21	Discuss the generalisability (external validity) of the study results	14	The study has provided a more concise method of measuring the degree of adherence to this treatment, has demonstrated the critical need for high levels of adherence to achieve adequate therapy effectiveness, and has identified the major factors that can influence this variable, serving as a starting point for other nurses to work on them.

**Other information**				
Funding	22	Give the source of funding and the role of the funders for the present study and, if applicable, for the original study on which the present article is based	-	No funding

*Give information separately for cases and controls in case-control studies and, if applicable, for exposed and unexposed groups in cohort and cross-sectional studies.

Note: An Explanation and Elaboration article discusses each checklist item and gives methodological background and published examples of transparent reporting. The STROBE checklist is best used in conjunction with this article (freely available on the Web sites of PLoS Medicine at http://www.plosmedicine.org/, Annals of Internal Medicine at http://www.annals.org/, and Epidemiology at http://www.epidem.com/). Information on the STROBE Initiative is available at www.strobe-statement.org.
